# Photovoltaic Electrochemically Driven Degradation of Calcon Dye with Simultaneous Green Hydrogen Production

**DOI:** 10.3390/ma15217445

**Published:** 2022-10-24

**Authors:** Jussara Câmara Cardozo, Djalma R. da Silva, Carlos A. Martínez-Huitle, Marco A. Quiroz, Elisama V. Dos Santos

**Affiliations:** 1 Institute of Chemistry, Federal University of Rio Grande do Norte, Lagoa Nova, Natal 59078-970, Brazil; 2 National Institute for Alternative Technologies of Detection, Toxicological Evaluation and Removal of Micropollutants and Radioactives (INCT-DATREM), UNESP, Araraquara 14800-900, Brazil; 3 School of Science and Technology, Federal University of Rio Grande do Norte, Lagoa Nova, Natal 59078-970, Brazil

**Keywords:** photovoltaic array, green hydrogen, electrochemical oxidation, dye, simultaneous processes

## Abstract

In this study, for the first time, the production of green hydrogen gas (H_2_) in the cathodic compartment, in concomitance with the electrochemical oxidation (EO) of an aqueous solution containing Calcon dye at the anodic compartment, was studied in a PEM-type electrochemical cell driven by a photovoltaic (PV) energy source. EO of Calcon was carried out on a Nb/BDD anode at different current densities (7.5, 15 and 30 mA cm^−2^), while a stainless steel (SS) cathode was used for green H_2_ production. The results of the analysis by UV-vis spectroscopy and total organic carbon (TOC) clearly showed that the electrochemical oxidation (EO) of the Calcon dye after 180 min of electrolysis time by applying 30 mA cm^−2^ reached up to 90% of degradation and 57% of TOC removal. Meanwhile, under these experimental conditions, a green H_2_ production greater than 0.9 L was achieved, with a Faradaic efficiency of 98%. The hybrid electrolysis strategy is particularly attractive in the context of a circular economy, as these can be coupled with the use of more complex water matrices to transform organic depollution into an energy resource to produce H_2_ as a chemical energy carrier.

## 1. Introduction

Hydrogen is considered as the central pillar of energy transformation and decarbonization, as it can contribute to the changes needed to reduce CO_2_ emissions [1,2], expanding energy transformation with the use of non-fossil energies while accentuating the international pressure to reduce CO_2_ emissions and comply with the Paris agreement [3,4]. H_2_ serves as a clean energy carrier and can be stored for later reconversion into electricity, as well as being used as a fuel or for carbon capture/recycling [5,6,7]. H_2_ production is frequently driven by renewable and non-renewable energy resources, such as fossil fuels— in particular steam methane reforming [8,9] and the NAFTA reform [10,11]—from biomass [12], and from biological sources [13,14,15,16]. Unfortunately, these H_2_ production methods are expensive, partially efficient, and environmentally polluting, as well as also producing low-purity H_2_ [17,18,19,20]. Meanwhile, the unique way to produce the cleanest H_2_ is from the well-known process of water electrolysis or, as commonly called, water splitting. However, the high energy expenditure is its main disadvantage. In this sense, the key to overcoming this economic obstacle is to supply the electrical energy for the electrochemical cell by using renewable energy sources, such as wind or solar power [17,18]. In Brazil, the abundance of resources that exceed demand, high solar irradiation, and consistent wind parks are a list of Brazil’s competitive advantages for producing green H_2_ using renewable energy [19,20].

Electrolytic water-splitting devices are an attractive technology for producing clean H_2_ which consist of stacks of electrochemical cells, in which the H_2_ evolution reaction (HER) and the oxygen evolution reaction (OER) take place at the cathode and anode compartments, respectively. Water electrolyzers are an established commercial technology; however, the use of other water matrices as resources for producing clean H_2_ remains limited.

Another way to offset the cost and safety problems for producing H_2_ from electrolysis would be a hybrid approach. On the contrary, to use clean water sources for anodic processes, the OER is replaced by an electrochemical organic oxidation reaction with aqueous effluents, which, according to environmental legislation, require treatment before their discharge. The most popular electrolytic process for removing organic pollutants from different water matrices is EO [21,22], which is highly efficient, versatile, and safe, as well as being considered an environmentally friendly technology when coupled to renewable energies [23]. 

Essentially, there are two different types of EO mechanisms: direct and indirect oxidation approaches [24,25]. Although direct oxidation is feasible under specific conditions, in some processes, indirect oxidation can become dominant when non-active electrodes (such as boron-doped diamond (BDD), PbO_2_, or Ti_4_O_7_) are used, because these are considered the most efficient anodes for oxidizing pollutants in water due to their high potential for the electrogeneration of a great amount of heterogenous free hydroxyl radicals (^•^OH) and/or other oxidants [26,27]. The ^•^OH effectively degrades the organic pollutants via redox reactions (electron transfer) and H_2_ atom abstraction, dissociating into a more biodegradable state, or can even be mineralized into the water, carbon dioxide and simple inorganic ions [28,29].

To enable the treatment of effluents and simultaneous generation of H_2_, the development of electrochemical cells where the anode and cathode are separated by cation exchange membranes (PEMEC) is necessary. This arrangement offers great advantages over the traditional single compartment, which is frequently used in EO, because proton exchange membranes, such as Nafion^®^, partially or totally delimit the transport of species such as particles, macromolecules, ions, gases, etc., letting only protons pass through, and exhibit high selectivity to H_2_ [30,31]. Thus, the hybrid process in a PEMEC, in which the EO of organic pollutants in the anodic semi-cell and the production of H_2_ in the cathodic semi-cell allows the collection of high-purity H_2_. It is important to remark that this hybrid method was initially reported several years ago; the pioneering investigations in this development can be credited to the work published by Lamy et. al. [32]. In this work, the use of organic compounds as sacrificial analytes stands out, which was shown to be a good alternative for splitting water to produce H_2_ because it reduces the amount of energy spent in the electrolysis process. However, the anodic compartment conditions are reduced to model-synthetic solutions.

Nowadays, hybrid electrolysis strategies are particularly attractive in the context of a circular economy as these can be coupled with the use of more complex water matrices to transform organic depollution into an energy resource to produce H_2_ as a chemical energy carrier. A recent and original example of this approach is the EO of Methyl Red with PbO_2_, BDD and Sb-doped SnO_2_ anodes [33] in a two-compartment electrochemical reactor with simultaneous H_2_ production in the cathode compartment [34]. Currently, some related studies by using a PEM-type cell have been recently published, oxidizing different sacrificial organic molecules to produce H_2_ [35,36,37]. 

The benefits of hybrid electrolysis are multifaceted to valorize low-value organics or waste, reaching significant technical impacts in the form of versatility, safety of operation, efficiency, and cost-effective technology, as well as integrating electrochemical-based solutions to fulfill Sustainable Development Goals (SDG 6 and 7) (i.e., depollution of water, sanitation, disinfection, water sustainability and energy security using green and modern energy sources) [38]. Afterwards, by replacing the OER with EO, the use of specific electric power conditions could constitute an increase in the effectiveness of both processes and a decrease on the component costs associated with membranes and electrocatalysts/electrode materials as well as electrolyzers. On the one hand, the key emerging technology in energy-water innovation solutions, due to the intensification of energy demands as well as worldwide water sustainability, is combining renewable energy sources with energy-efficient water treatment methods.

In this context, efficient energy–water solutions, such as electrochemical H_2_ production and EO, can be incorporated with renewable energy.

Therefore, the objective of this work is to demonstrate that H_2_ is efficiently produced simultaneously with the EO of Calcon azo dye, used as a sacrificial analyte with a split electrochemical flow cell, featuring a BDD electrode as an anode and two types of cathodes. The technology proposed here uses a photovoltaic array as an energy source to drive the operation of the designed PEM-type cell, establishing a promising, efficient, and sustainable alternative to producing green H_2_ during the decontamination of water to fulfill SDG 6 [38].

## 2. Materials and Methods

All model solutions were prepared with ultrapure water obtained using a Millipore Milli-Q purification (≈18.0 MΩ) system at 25 °C. Chemicals were of the highest quality commercially available and used without further purification. Sulfuric acid (H_2_SO_4_) and sodium sulfate (Na_2_SO_4_) were supplied by Dinámica Química LTDA. Calcon dye (Eriochrome Blue Black R) was supplied by Hetero Drugs Limited as a solid dark blue product of analytical grade and used without any pretreatment; the chemical data and characteristics of the azo group are summarized in Table 1.

### 2.1. Electrochemical Systems

**Figure 1:**
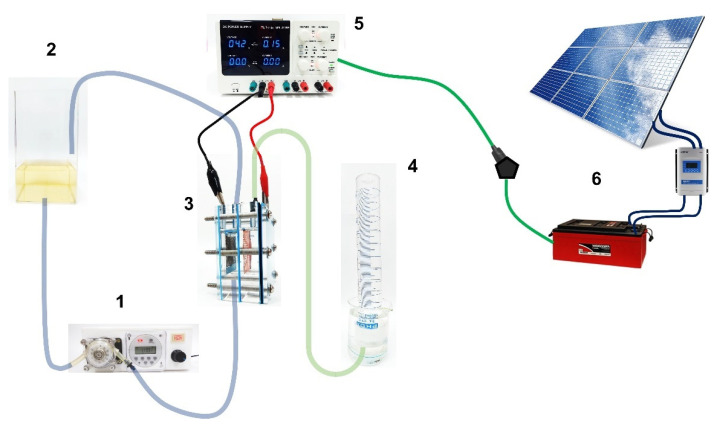
Diagram of the complete electrochemical system, including the photovoltaic power module: (1) peristaltic pump, (2) Calcon dye solution reservoir (1 L), (3) electrochemical cell PEM-type, (4) H_2_ collector, (5) power supply, (6) solar photovoltaic (PV)-battery system.

The electrochemical reactor consisted of two electrodes of dimensions (15.0 cm^2^ BDD and 18.2 cm^2^ SS), protected inside a (10.0 × 7.5 × 1.7 cm) acrylic case, with pre-drilled holes for solution inlet, outlet, and for electrical connections. The two-compartment cell was separated by a Nafion^®^ membrane of 350-type with the opaque face towards the catholyte solution (well-known as a PEM cell). Each compartment has a volume of 0.04 L with a gap of 3.4 cm between electrodes. Electrolysis of 1 L of synthetic solution containing 20 mg L^−1^ of Calcon dye in 0.25 M H_2_SO_4_ as the supporting electrolyte was performed by applying 7.5, 15 and 30 mA cm^−2^ for 180 min in a thermoregulated glass tank at 25 °C and circulated through the cell by a peristaltic pump at a constant flow rate of 39 mL min^−1^. The H_2_ produced at the cathodic reservoir, in concomitance with the oxidation of the Calcon dye at the anodic compartment, was collected over distilled water (Figure 1), and the volume measured by means of a 1 L inverted burette directly connected to the cathode compartment. An Nb/BDD electrode was used as an anode, while as a cathode, a 316-type stainless-steel (SS) plate or a Ni-Fe-based SS mesh were used. Both shapes had the same geometric dimensions but differed in the exposed area and type of steel (in the supplementary material (SM), see Figure S1 for Ni-Fe-based SS mesh). Additionally, the effect of the Calcon concentration (20 and 50 mg L^−1^) and the flow rate (10, 39 and 90 mL min^−1^) in the anodic compartment on the production of green H_2_ were evaluated.

### 2.2. Solar PV-Battery System

Electrochemical approaches were controlled by two polycrystalline silicon solar PV modules (Canadian CS6U-325p) connected in series with a total peak power of 640 Wp. The PV modules were placed on the roof of the Nucleo of Studies in Petroleum and Renewable Energies (NUPER) at the Federal University of Rio Grande do Norte, Natal, Brazil (W 350 12′, S 050 54′), tilted at 5° and at a south orientation (20° W). A maximum power point tracker (Victron Blue solar 150/45-MC-4) was connected in between PV modules and the two 12 V batteries (Solar Freedom, 12 V/240 Ah each) to guarantee that the maximum electric energy available was always extracted from the solar cells. The power supply to regulate the current applied to the electrochemical treatment reactor, and the potentiostat/galvanostat used for the electrochemical device, were directly connected to the batteries, as described in a previous work [39,40].

### 2.3. Apparatus and Analytical Procedures

The electrochemical characteristics of the Calcon oxidation were obtained in a typical three-electrode cell using an AUTOLAB model PGSTAT302N potentiostat/galvanostat controlled by GPES (General Purpose Electrochemical System) software. The working electrode was Nb/BDD, the auxiliary electrode a Pt wire, and an Ag/AgCl (3 M KCl) electrode was used as reference, respectively. Cyclic voltammetry (CV) experiments were carried out in 0.25 M H_2_SO_4_ as the supporting electrolyte, containing 20 mg L^−1^ of dye concentration. The effect of the scan rate (5–100 mV s^−1^) and dye concentration (1.5–100 mg L^−1^) was evaluated by cyclic voltammetric analysis. The color removal was monitored by the decrease in absorbance measured at 513 nm [41], such that the percentage of discoloration of the solution during electrolysis was estimated from the following Equation (1):

(1) $$Color\;removal\;\left(\%\right)=\frac{A_{0}-A_{t}}{A_{0}}\times 100$$

where *A_0_* is the initial absorbance and *A_t_* the absorbance at the time *t*. The absorbance was determined using an UV–vis spectrophotometer Analytikjena Specord 210 Plus and an open quartz cell of 0.5 mL and optical length of 1 cm.

The degree of mineralization of the Calcon dye in solution was followed by a decrease in the total organic carbon (TOC) that was measured with a MULTI N/C 3100 of Analytik Jena according to the ASTM D 7573-18 standard test method. Meanwhile, the H_2_ produced at the cathodic compartment was collected using an inverted burette with defined temperature and pressure conditions and considering the vapor pressure of water to calculate the actual volume of H_2_ generated [32,33]. The characterization of the Calcon solution, after and at the end of the electrolysis, was performed by the Fourier transform infrared spectroscopy (FTIR) and the gas chromatography coupled with mass spectrometry (GC-MS). A PerkinElmer Frontier was used for FTIR analyses with a scan rate of 400–4000 cm^−1^, and the samples were deposited on the aluminum surface mirror obtained with a 3D-printed fixture lab home-made for ex situ measurements of specular reflectance [42]. The samples were chosen at the best experimental condition using Calcon 20 mg L^−1^ in 0.25 M H_2_SO_4_ at a current density of 30 mA cm^−2^. For the study with GC-MS, a Shimadzu QP2010 SE model equipped with a 30 m long RESTEK-RTX-5MS capillary column (0.25 mm film thickness and 0.25 mm internal diameter) and a quadrupole mass detector was used. The acquisition time was 19 min, and the mass range was 35 to 500 m/z. Helium was used as the carrier gas. At the end of the same electrolysis analyzed by FTIR, the concentration of five carboxylic acids (acetic, formic, fumaric, maleic and oxalic) was accessed by high-performance liquid chromatography (HPLC) analyses in a Thermo Scientific™ (Waltham, MA, USA) DIONEX^TM^ system (LC Ultimate 3000) with a diode array detector (Ultimate 3000 DAD). A volume of 10 µL of sample was injected by an Ultimate 3000 autosampler. The compounds were determined by an Acclaim Organic Acids column (Acclaim OA, 5 mm, 120 Å, 4.0 × 250 mm), which was used at 25 °C. The mobile phase consisted of 100 mM of Na_2_SO_4_, pH 2.65 (adjusted with methanesulfonic acid), which was eluted at 600 mL min^−1^ for 15 min. Retention times were 3.3, 3.8, 4.4, 5.6, and 7.0 min for oxalic, formic, acetic, malic and fumaric acids, respectively. Some experiments were randomly run in duplicate, and the withdrawn samples were analyzed in duplicate to minimize the experimental error. Deviations between runs were always lower than 5% for all determinations. pH conditions in cathodic and anodic reservoirs were also monitored during the hybrid process by using a HANNA pHmeter HI1131B. 

## 3. Results

### 3.1. Voltametric Behavior of Calcon Dye on BDD Surface

**Figure 2:**
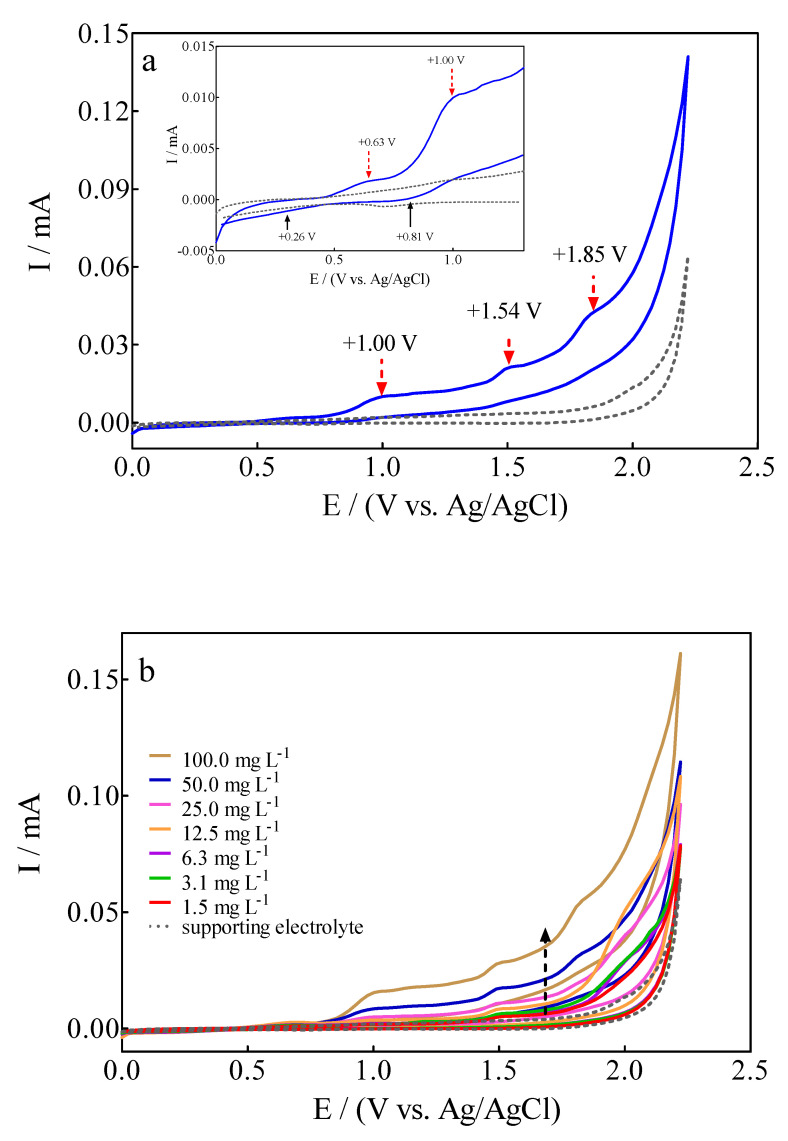
(**a**) Cyclic voltammograms for (▬) 100 mg L^−1^ Calcon + 0.25 M H_2_SO_4_ solution and (**---**) supporting electrolyte on BDD electrode at 50 mVs^−1^ in the potential range from 0.0 V to +2.3 V (vs Ag/AgCl (3M KCl)). (**b**) Electrochemical behavior of anodic and cathodic peaks at the CV profiles, as a function of Calcon dye concentration (1.5–100 mg L^−1^), at 25 °C.

The electrochemical behavior of 100 mg L^−1^ Calcon + 0.25 M H_2_SO_4_ solution on a BDD electrode was examined by cyclic voltammetry (CV) at 50 mV s^−1^, as is shown in Figure 2a. The voltametric profile registered interesting signals where, on the one hand, the presence of anodic peaks could be associated with different (reversible and/or irreversible) Calcon dye oxidations stages on the diamond surface, while on the other hand, few cathodic peaks were observed in the reverse potential scan within the CV potentials range.

The four observed anodic peaks were located at +0.63 V (E_pa_1), +1.00 V (E_pa_2), +1.54 V (E_pa_3) and +1.85 V (E_pa_4), while only two cathodic peaks were detected at +0.26 V (E_pc_1) and +0.81 V (E_pc_2). The CV profile clearly evidenced the versatile behavior of the BDD electrode due to the effective promotion of direct and indirect oxidation routes of the azo dye at its surface [43]. Anodic peaks (E_pa_1 = +0.63 V and E_pa_2 = +1.00 V) at the lower overpotential region (<1.6 V) are associated with direct electron-transfer on the diamond surface, while the current-voltammetric signals (E_pa_3 = +1.54 V and E_pa_4 = +1.85 V) at the high overpotential region (>1.62 V) should be related to the indirect oxidation approach via the participation of free heterogeneous ^•^OH (which are electrogenerated via water discharge (Equation (2)) in the Nernst layer (commonly named “reaction cage” [44,45]).

BDD + H_2_O → BDD(^•^OH) + H^+^ + e^−^ (2)

In the former process, the cathodic peaks, E_pc_1 = +0.26 V and E_pc_2 = +0.81 V, seem to correspond to reversible oxidation behaviors achieved at the lower overpotential region (<1.6 V). Meanwhile, no cathodic peaks were registered at the high overpotential region in the latter approach, which corresponds to the indirect oxidation mechanism. This electric-diamond surface feature is interesting because it confirms that the EO of organics does not only depend on the general rule for active and non-active anode classification [26,43,46]. Instead, the EO also depends on the chemical structure of the pollutants, the properties of diamond film, and the electrolyte and its concentration, as well as the potential or current in which the EO is promoted. In fact, similar assertions were recently demonstrated and published by Cardozo et al. [46] when olanzapine (a pharmaceutical drug) was electrochemically oxidized on the BDD surface where a detailed investigation was performed to understand, from a fundamental electrochemistry point of view, the versatility of actual diamond surfaces.

In the case of Calcon dye, when the voltametric profile was analyzed, as a function of its concentration, it was observed that the peak current for the all-anodic signals increased linearly as a function of the concentration, and these voltammetric signals were slightly shifted towards more positive potentials (Figure 2b). An interesting feature is that a decrease in the current signals for the cathodic peaks, E_pc_1 = +0.26 V and E_pc_2 = +0.81 V, was also achieved when an increase on the Calcon concentration was attained. This behavior also seems to decrease their intensity in terms of the reversibility [46]. Consequently, this effect on anodic and cathodic peaks, sems to originate from the influence of Calcon mass transport towards the BDD surface, which can be examined by means of the relationship of current intensity or peak potential as a function of scan rate.

**Figure 3:**
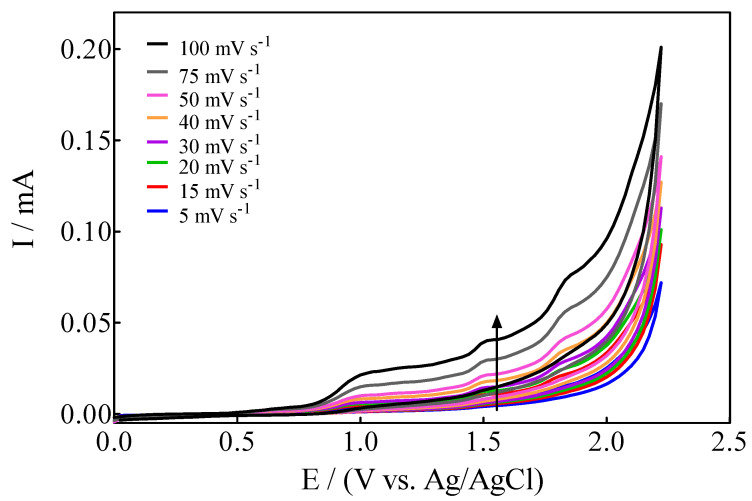
Cyclic voltammograms of Calcon (100 mg L^−1^) on BDD in 0.25 M H_2_SO_4_ as a function of the scan rate from 5 to 100 mV s^−1^.

Based on the results reported above, another important aspect to be investigated is the variation of the intensity of anodic current (i_p_) as a function of the scan rate, from 5 to 100 mV s^−1^ (Figure 3). 

**Figure 4:**
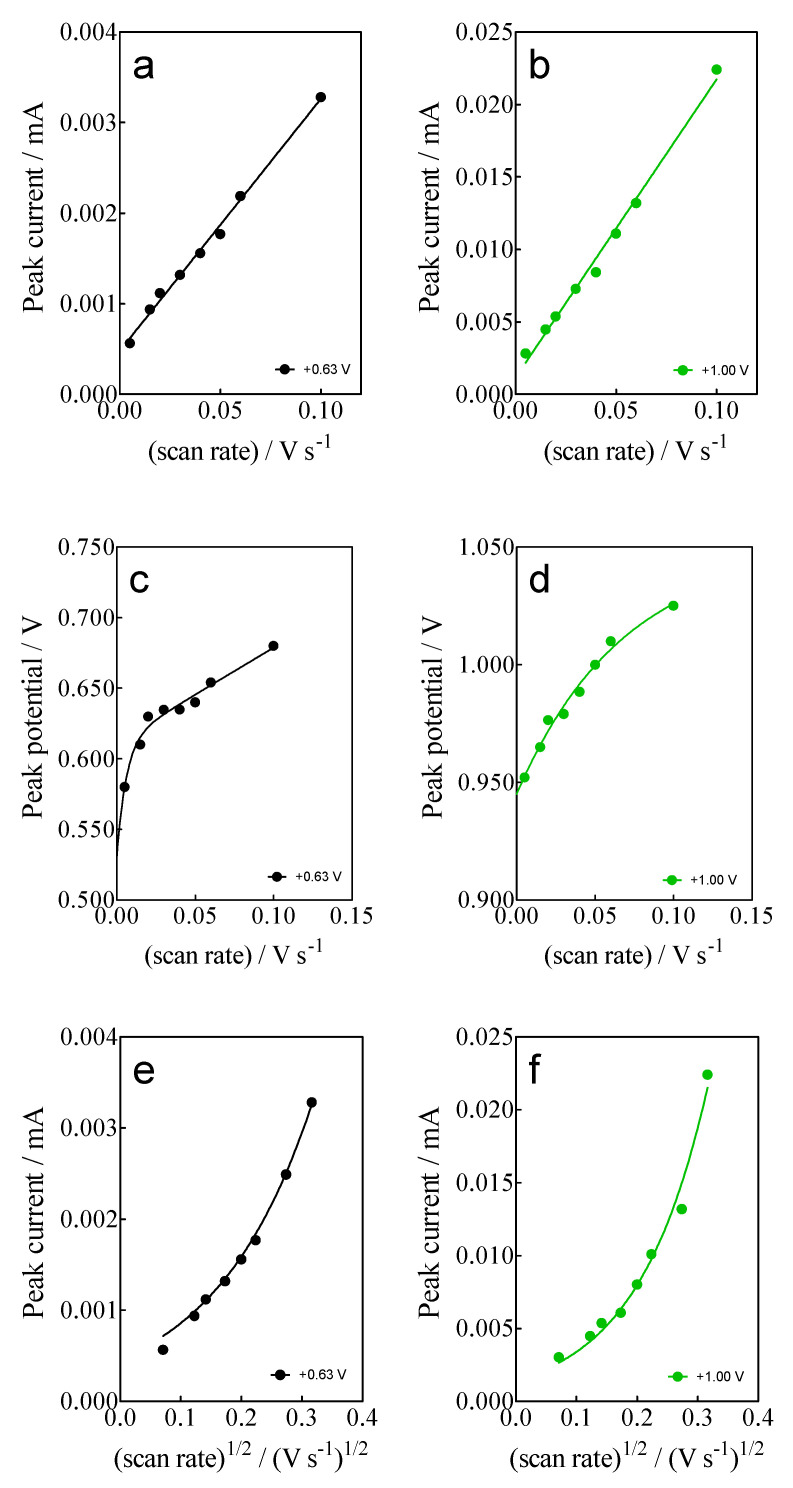
(**a**,**b**) Linear dependence of peak current (*i_p_*) at +0.63 V (E_pa_1) and (+1.00 V (E_pa_2) with the potential scan rate (V s^−1^); (**c**,**d**) Non-linear dependence of E_pa_1 (+0.63 V) and E_pa_2 (+1.00 V) on the ν (V s^−1^); and (**e**,**f**) relationship between the *i_p_*, for E_pa_1 (+0.63 V) and E_pa_2 (+1.00 V), and the square root of the scan rate (𝑣^1/2^). All plots were obtained for the Calcon dye (100 mg L^−1^) in 0.25 M H_2_SO_4_ at the BDD electrode.

**Figure 5:**
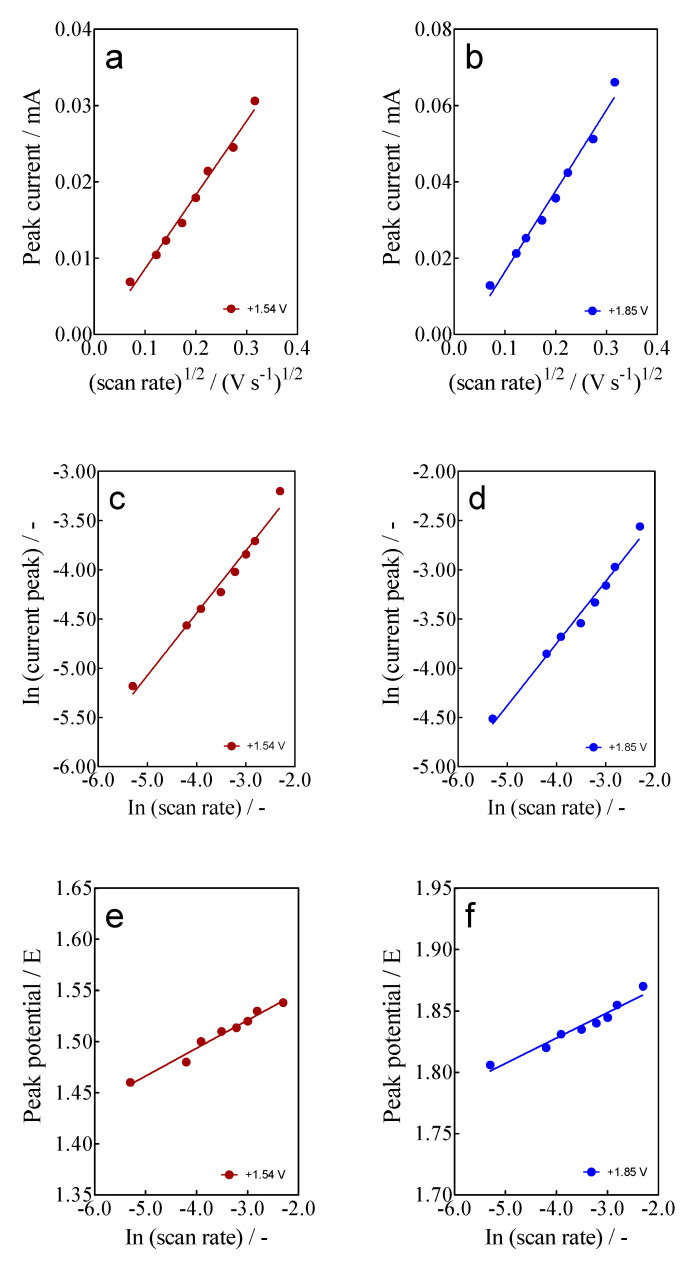
Linear dependence of the *i_p_* at E_pa_3 (+1.54 V) and E_pa_4 (+1.85 V) with: (**a**,**b**) square root (𝑣^1/2^), (**c**,**d**) scan rate (𝑣) of potential (as ln both), and (**e**,**f**) peak potential E_pa_3 (+1.54 V) and E_pa_4 (+1.85 V) on the ln ν (V s^−1^) for the Calcon dye (100 mg L^−1^) in 0.25 M H_2_SO_4_ at the BDD electrode.

At this point, two effects were observed:

(i) For anodic peaks at the lower overpotential region (<1.6 V), E_pa_1 (+0.63 V) and E_pa_2 (+1.00 V), a linear relationship of the I_p_ (for both peaks) with the scan rate, according to the Randles–Sevcik model [46], is attained, suggesting an adsorption step of Calcon on the BDD surface (Figure 4a,b). This is also confirmed by the non-linear dependence on the E_pa_1 and E_pa_2 vs. scan rate (Figure 4c) as well as on the I_p_ (for both peaks) with the square root of the scan rate (Figure 4e,f), as shown in Figure 4. Therefore, a direct interaction of the Calcon structure could be attained at E_pa_1 (+0.63 V) and E_pa_2 (+1.00 V) via an electron-transfer reaction as the first step of the oxidation process [46].
(ii) For the current-voltammetric signals, identified as E_pa_3 (+1.54 V) and E_pa_4 (+1.85 V), the Randles–Sevcik model was not followed (I_p_ vs. scan rate), indicating that the non-dependency on the direct-electron transfer reactions is attained at the high overpotential region [46]. Although the displacement of the peak potentials was not significant for these anodic peaks (Figure 3), the increase in the intensity of the current peaks (*i_p_*) with the square root of the scan rate (𝑣^1/2^) obeys the criterion established by Bard and Faulkner [46,47] for an irreversible process (Figure 5a,b), through the following Equation (3) in the range of 5 to 100 mV s^−1^ (Figure 5):

(3) $$i_{p}=\left(2.99\times 10^{5}\right)\alpha^{1/2}AC_{0}^{*}D_{0}^{1/2}\upsilon^{1/2}$$

where *i_p_* represents the peak current (A), *α* is the transfer coefficient (dimensionless), *A* is the surface area of the electrode (cm^2^), C_0_^*^ is the bulk initial concentration of Calcon (mol L^−1^), *D_0_*^1/2^ is the diffusion coefficient (cm^2^ s^−1^) and ν is the linear scan rate (V s^−1^). These results confirm the fact that the Calcon oxidation on BDD in 0.25 M H_2_SO_4_ is a process of a typically irreversible nature [48,49], where the observed voltammetric behavior occurs by diffusion factors [50], such as slow electron transfer, and higher ohmic resistance at the electrode/electrolyte interface. Additionally, if the behavior of the *i_p_* is analyzed with the potential scan rate, both in their logarithmic form (R^2^ = 0.992), a linear relationship is achieved again, with slopes equal to 0.53 and 0.55 (Figure 5b,c) for E_pa_3 (+1.54 V) and E_pa_4 (+1.85 V), suggesting that the electrochemical reaction of Calcon dye at E_pa_3 and E_pa_4 is influenced by the diffusion behaviors. Finally, as can be observed in Figure 5e,f, the variation of the peak potential at E_pa_3 and E_pa_4, as a function of ln(ν), also exhibited a linear relationship (r^2^ = 0.9812 and 0.9913, respectively) with slopes ranging from 0.0274 and 0.0211 V, confirming that these processes are irreversible, as reported by Sanati et al. [51]. 

It is possible to prove and comprehend that these two processes can be accomplished simultaneously at the BDD electrode during Calcon EO based on the experimental and mathematical electrochemical analysis. Firstly, a direct electron transfer occurs at lower potentials, triggering a reversible reaction that converts Calcon into two possible by-products, which is then reduced to release the Calcon again. This mechanism favors a partial adsorption stage due to the exitance of an interaction on the BDD surface [43,46]. The chemical structure of the target organic compound, the electrical density of the functional groups, and the surface characteristics of the diamond electrode may all play a role in this interaction, as already shown by other authors [43,52]. A facilitated oxidation process is preferred at higher positive potentials where the ^•^OH radicals can be electrogenerated [26]. In fact, this process depends on the operating conditions (such as the electrochemical reactor, dye concentration, temperature, supporting electrolyte and so on), hydroxyl radical concentration, organic matter mass transport control, and chemical oxidation interactions between dye and oxidants [53]. For these reasons, bulk electrochemical Calcon oxidation experiments should also be investigated to understand the varied behavior of diamond electrodes.

### 3.2. UV-Vis Spectroscopic Characteristics of Calcon in Aqueous Media

**Figure 6:**
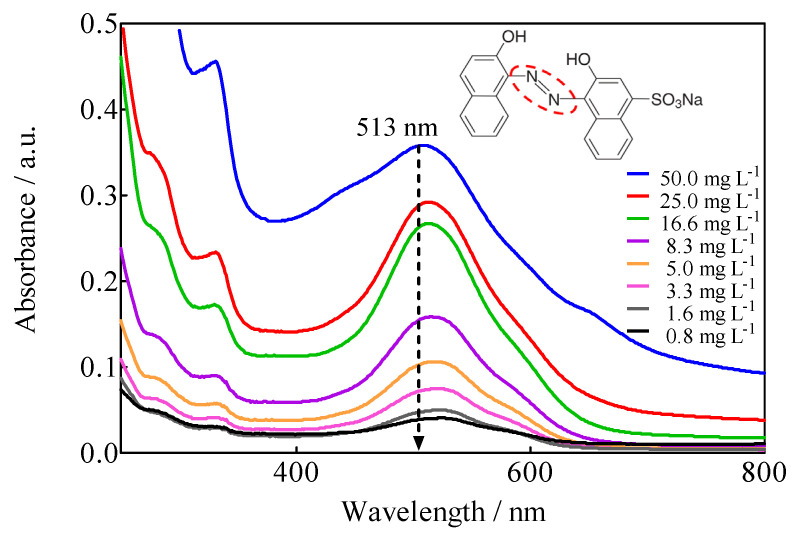
UV-vis absorption spectra, in adimensional units of absorbance, of 0.25 M H_2_SO_4_ + *x* mg L^−1^ Calcon solutions as a function of Calcon concentration, 0 < *x* ≤ 20 mg L^−1^.

The UV-Vis spectrum of the 0.25 M H_2_SO_4_ + 50 mg L^−1^ M Calcon solution exhibited a complex profile, since, as observed in Figure 6, it presents absorption bands both in the UV region (<400 nm) and in the visible region (>400 nm). The former is associated with the aromatic structure of the molecule (‘B, ‘L_a_ and benzenoid bands), while the broad and well-defined band at 513 nm is related to the azo group (-N=N-) characteristic of the dye. Although the UV bands are intense, only the ‘B band at 226 nm is defined, even when it is of little use because it is not specific. In the meantime, the band at 513 nm, although of low intensity, is specific to the azo group and, therefore, responsible for the color of the solution. This quality allowed monitoring the dye removal through the discoloration of the solution. Applying Beer’s law (*A* vs. [Calcon] with R^2^ = 0.9966) to the absorption band at 513 nm of Figure 6, the molar absorptivity coefficient (ε_513_) was estimated to be: ε_513_ = 6.08 × 10^3^ L mol^−1^ cm^−1^, which is a suitable value for quantitative purposes.

### 3.3. Electrochemical Oxidation of Calcon 

**Figure 7:**
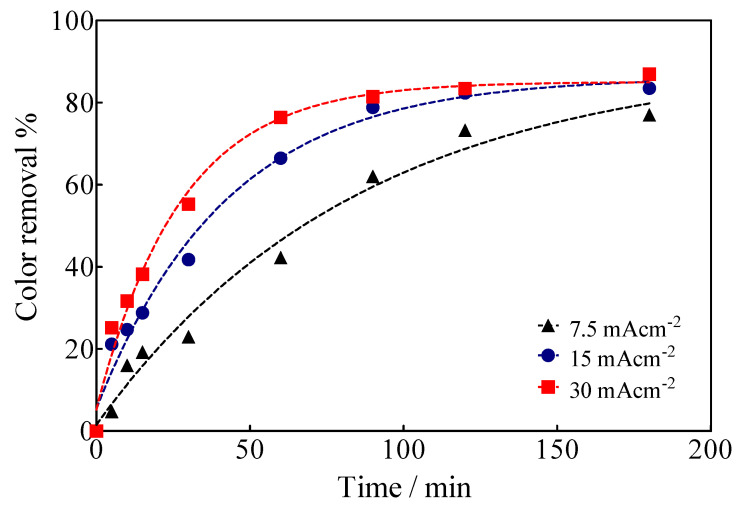
Color removal efficiency as a function of the electrolysis time and the applied current density for the EO of Calcon at BDD anodes in 0.25 M H_2_SO_4_.

EO of Calcon was carried out under galvanostatic conditions in a cell with two compartments separated by a Nafion^®^ membrane with 0.04 L capacity in each compartment under flow conditions (2 mL min^−1^ by means of a peristaltic pump (Figure 1), using a BDD electrode (15 cm^2^) as an anode, while a SS plate or a Ni-Fe based SS mesh were used as cathodes. The color removal of Calcon was monitored using UV-vis spectroscopy characterized by an absorbance maximum at a wavelength of 513 nm (Figure 6), as a function of the applied current density as well as the electrolysis time, such as is shown in Figure 7. 

The decrease of the absorption band of Calcon solution in the UV-vis spectra at 513 nm indicated the fragmentation of the azo group. This result also agrees with the outcomes obtained by FTIR analysis (see Figure S2), demonstrating that the cleavage of the azo bonds was achieved via the decrease of the peak that corresponds to the azo vibration at 1700 cm^−1^. Additionally, FTIR spectra exhibited variations on the peak width and position associated with the O-H stretching at 3694 cm^−1^, indicating that electron distribution in the molecular bond had changed and demonstrating that the chemical structure of the Calcon was modified. For the peak position related to the vibrations from 1400 cm^−1^ to 1600 cm^−1^, as well as the C-N stretching at 1200 cm^−1^, and -SO stretching at 1116 cm^−1^, similar behavior was also observed. Lastly, the peaks observed at the FTIR spectra showed that no *cis*- or *trans*- Calcon predominance was attained in the solution, which could influence the degradation of starting pollutant concentration in the solution. 

Based on the spectrophotometric analysis, up to 80% decolorization was achieved at 15 and 30 mA cm^−2^ after 180 min of electrolysis time, while at 7.5 mA cm^−2^ and the same electrolysis time, up to 70% of the solution was decolorized. This behavior is associated with highly efficient electrocatalytic BDD features for the electrochemical production of oxidizing species [54], especially ^•^OH radicals that are formed by water discharge and remain physisorbed on the anode surface [27,53], provoking the fragmentation of the chemical structure of Calcon in solution. However, when the electrolysis process is carried out in the sulfate medium (H_2_SO_4_ and/or Na_2_SO_4_), the formation of secondary oxidizing species, such as SO_4_^−•^ and S_2_O_8_^2−^ [54,55,56,57], also contributes to the general oxidation process (degradation of Calcon chemical structure and its intermediates). A chemical scheme (4) of the formation of these oxidizing agents is shown below.

(4) BDD+H2O →BDD(O•H)+H++e−BDD(O•H)+SO4−2 →BDD(SO4•−)+OH−BDD(SO4•−)+SO4−2 →S2O8−2+e−}

In the case of SO_4_^−•^, these species have excellent qualities as oxidizing agents, since their greater oxidation potential (2.5–3.1 V) with respect to ^•^OH radicals (1.8–2.7 V) and their ability to act over a wide pH range gives them high activity for the degradation of organic pollutants [56,57,58]. Therefore, the color removal of the Calcon solution is completely associated with the increase in the production of these oxidizing agents by increasing the current density applied to the electrochemical cell. In fact, the anolyte remained with acidic pH conditions (pH around 0.9) during 120 min of electrolysis, favoring the same chemical dye structure in the solution and the availability of dissolved sulfate ions to promote the electrogeneration of sulfate-based oxidizing species. In accordance with the above, the degradation and elimination of Calcon dye at the different applied current densities (7.5, 15 and 30 mA cm^−2^) was analyzed by measuring the TOC, a parameter that indicates the mineralization performance of the organic compound in solution, based on the chemical Equation (5):

C_20_H_13_N_2_NaO_5_S + 39 H_2_O → 20 CO_2_ + 2 NH_4_^+^ + Na^+^ + SO_4_^−2^ + 83 H^+^ + 84 e^−^ (5)

**Figure 8:**
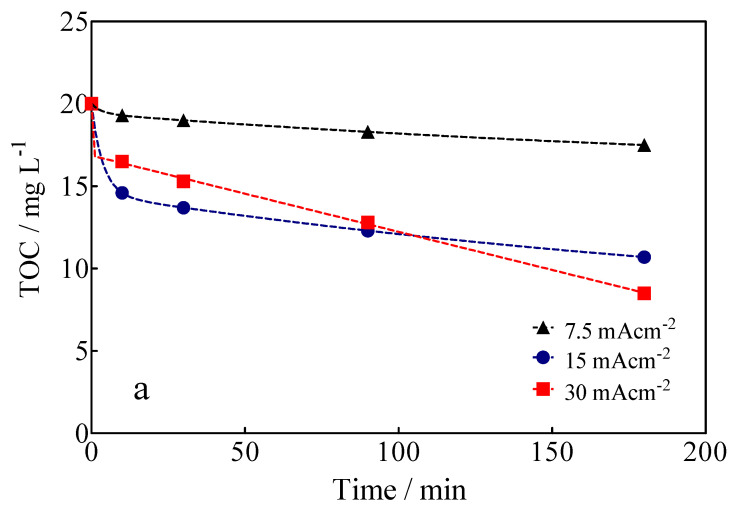

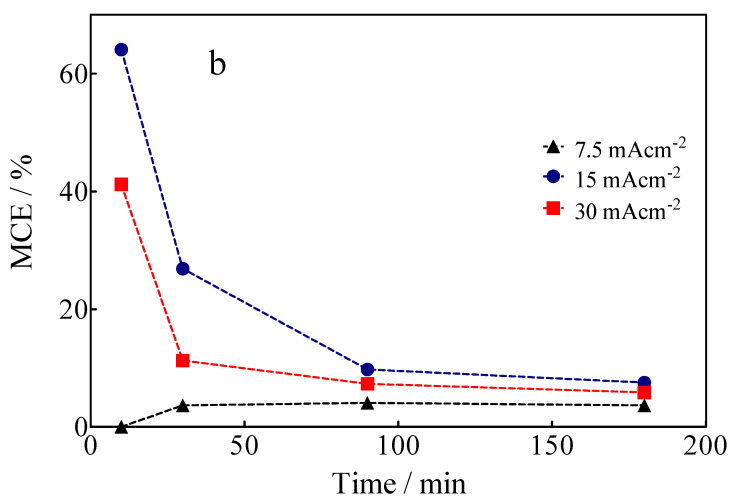
(**a**) Variation of TOC as a function of the electrolysis time and applied current density for the EO of Calcon dye at the BDD anode in 0.25 M H_2_SO_4_. (**b**) MCE for Calcon dye as estimated from the values of TOC measured for the electrolysis process.

The variation of TOC, as a function of the applied current density and electrolysis time, is shown in Figure 8a. The data in Figure 8a show that 58% of mineralization of the Calcon dye was achieved at 30 mA cm^−2^ and 180 min of electrolysis time, but only 9.3% and 41% were reached at 7.5 and 15 mA cm^−2^, respectively, under the same experimental conditions. These results show that the Calcon dye can be oxidized in solution, but also that byproducts generated are still recalcitrant, requiring long electrolysis times to achieve complete electrochemical incineration to CO_2_ and H_2_O. From the TOC data, in its ∆(TOC)_exp_ form, as well as the chemical equation of mineralization of the Calcon dye, in Equation (5), it is possible to estimate the mineralization current efficiency (MCE), according to Equation (6) [58]:

(6) $$MCE\left(\%\right)=\frac{nFV\left(TOC_{0}-TOC_{t}\right)}{4.32\times 10^{7}mit}\times 100$$

where *TOC_0_* and *TOC_t_* are the initial and time values of the *TOC* (in mg C L^−1^), *m* is the number of carbon atoms contained in the Calcon molecule and converted in CO_2_ according to the chemical Equation (5), *F* is the Faraday constant (96,487 C mol^−1^), *V* is the volume of electrolyzed solution (in L), *i* is the intensity of the applied current (in A), *n* is the number of electrons involved in the overall mineralization of Calcon dye as shown in chemical Equation (5), *t* is the electrolysis time (in h), and 4.32 × 10^7^ is a conversion factor (= 3600 s h^−1^ × 12,000 mg C mol^−1^).

The results of MCE for BDD-anodic electrolysis are shown in Figure 8b. As can be observed, both the degree of mineralization (∆TOC) and the percentage of mineralization efficiency (MCE) are low at low applied current densities (≤7.5 mA cm^−2^); this fact can be attributed to the direct oxidation of Calcon (with dye adsorption on diamond film without deactivation of its surface (as demonstrated by electrochemical measurements)) and, since the electrolysis conditions are not the best to produce sufficient oxidation agents (^•^OH, SO_4_^−•^ and S_2_O_8_^2−^) that promote indirect oxidation [27,59], it is assumed that the direct oxidation process is promoted by the interaction of the BDD electrode surface with unpaired electrons from the nitrogen atoms present in the azo group (-N=N-) [59]. Conversely, at high applied current densities (>7.5 mA cm^−2^) and electrolysis times, the level of mineralization significantly increases (Figure 8a). However, the formation of intermediates that are difficult to mineralize occurs, as well as the oxygen evolution reaction (OER) becoming more important, influencing the complete elimination of organics from the solution [60,61] and, consequently, the mineralization efficiency of the process (Figure 8b) [46].

**Figure 9:**
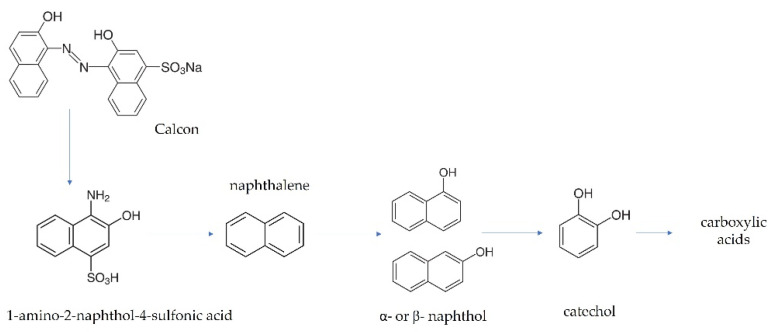
Proposed pathway for the EO of Calcon at BDD anodes in 0.25 M H_2_SO_4_.

Evaluating the production of intermediates by GC-MS and HPLC when the electrolysis of a solution of Calcon 20 mg L^−1^ in 0.25 M H_2_SO_4_ at 30 mA cm^−2^ was performed, the GC-MS spectra allowed us to identify some degradation products that probably correspond to the intermediates of naphthalene (*m*/*z* = 128, 102, 64, 51), ethyl salicylic acid (*m*/*z* = 166, 120, 92, 65), 1- or 2-naphthol (*m*/*z* = 144, 115, 89, 72) and catechol (*m*/*z* = 110, 81). Meanwhile, the HPLC results demonstrated that the evolution of five carboxylic acids was attained, as shown in Figure S3. Exploring the intermediates formed during Calcon electrochemical degradation, it is evident that the oxidizing species attacked mainly the chromophore group, the double bonds, and aromatic rings in the dye chemical structure, generating intermediates and carboxylic acids, as illustrated in the oxidation pathway proposed (see Figure 9). These results agree with the spectrophotometric and FTIR analysis as well as the degradation and mineralization outcomes that demonstrated, on the one hand, the rapid decay of the color, and on the other hand, the partial mineralization of the organic matter in the solution, respectively.

In recent years, there has been a significant effort to increase the sustainability not only of industrial but also of environmental processes. New concepts arising from both the theory of circular economy and the application of life cycle assessment tools have prepared researchers and technicians for a change of paradigm in electrochemically assisted waste remediation technologies. In this sense, the new electrochemical concept, which focuses on substituting destructive technologies or in developing hybrid processes, has until now searched for the treatment of waste in both cases for electrochemical technologies forthcoming to obtain highly added-value products, or brick molecules from the pollutants, instead of favoring their mineralization during the treatment. Within this framework, several innovative electrochemical approaches are being developed as hybrid processes where advanced materials science may help in achieving sustainable development goals (SDG) in the key areas of clean water and sanitation (SDG 6) and affordable and clean energy (SDG 7). Following the replacement of the OER with EO (as the desired anodic process) in conventional water splitting by the employment of particular electric power conditions, this may result in an improvement in the efficiency of hybrid processes to produce high added-value products as well as to overcome significant drawbacks, such as the reduction of the component costs, electrocatalysts/electrode materials, and electrolyzers. This is the case in the production of green H_2_. 

### 3.4. Green H_2_ Production

In the previous section, the electrochemical discoloration of the Calcon dye was almost 85%, and the mineralization practically 60% at the BDD anode in 0.25 M Na_2_SO_4_ by applying 30 mA cm^−2^ in electrolysis time at 180 min. The new circular economy paradigm “from waste to resource” is the target, and this means the necessity of maximizing resource recovery in a safe and sustainable way, which is going to become mandatory in further research related to waste and wastewater treatment. Therefore, the amount of H_2_ produced in the cathodic compartment was determined simultaneously with the occurrence of the EO of Calcon in the anodic compartment during a new set of experiments. For these experiments, the chosen anolyte was a 20 mg L^−1^ Calcon solution in 0.25 M Na_2_SO_4_, while the catholyte was only a 0.25 M H_2_SO_4_ solution. According to Figure 1, both compartments were separated by a Nafion^®^-350 membrane. The materials chosen as cathode were an SS plate or a Ni-Fe based SS mesh. Meanwhile, the anodic compartment was assembled with a BDD anode. 

**Figure 10:**
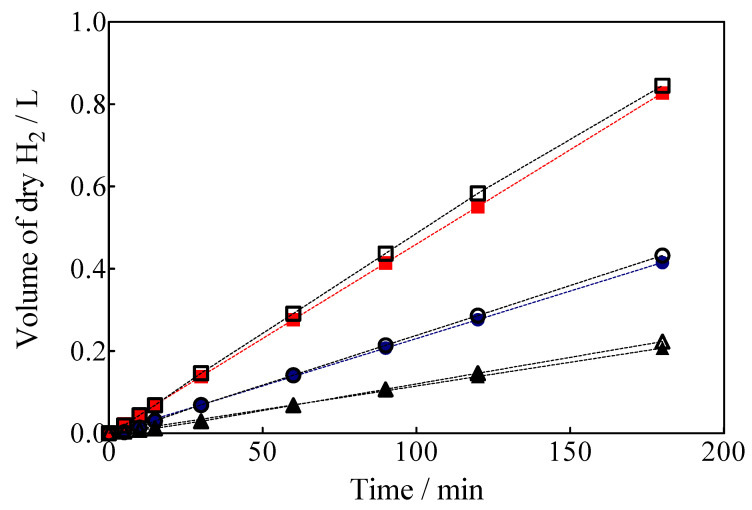
Volume of green H_2_ produced in a hybrid process with simultaneous Calcon EO in the anodic compartment with the BDD anode, applying (▲, ∆) 7.5 mA cm^−2^, (●, ○) 15 mA cm^−2^ and (■,☐) 30 mA cm^−2^. Empty circles: theoretical values; solid circles: experimental values.

Another important feature is that the complete electrochemical system is powered by a photovoltaic array; therefore, the H_2_ produced using renewable energy sources is denominated as green H_2_ [1]. Green H_2_ production results by using a hybrid approach through applying 7.5, 15 and 30 mA cm^−2^ are shown in Figure 10, as a function of electrolysis time which were estimated from Equation (7) [62]:

(7) $$V_{th}=\frac{k_{e}\times i\;\times t}{\rho }$$

Where *ke* = (*M*/*n*F) is the electrochemical equivalent (kg A^−1^ s^−1^), *n* is the electron number (2 for H_2_), *F* is the Faraday constant (96,487 C mol^−1^), *M* is the molar mass of H_2_ (Kg mol^−1^), *i* is the intensity of applied total current (A), *t* is the electrolysis time (s), and *ρ* is the H_2_ gas density (0.0818 kg m^−3^).

**Figure 11:**
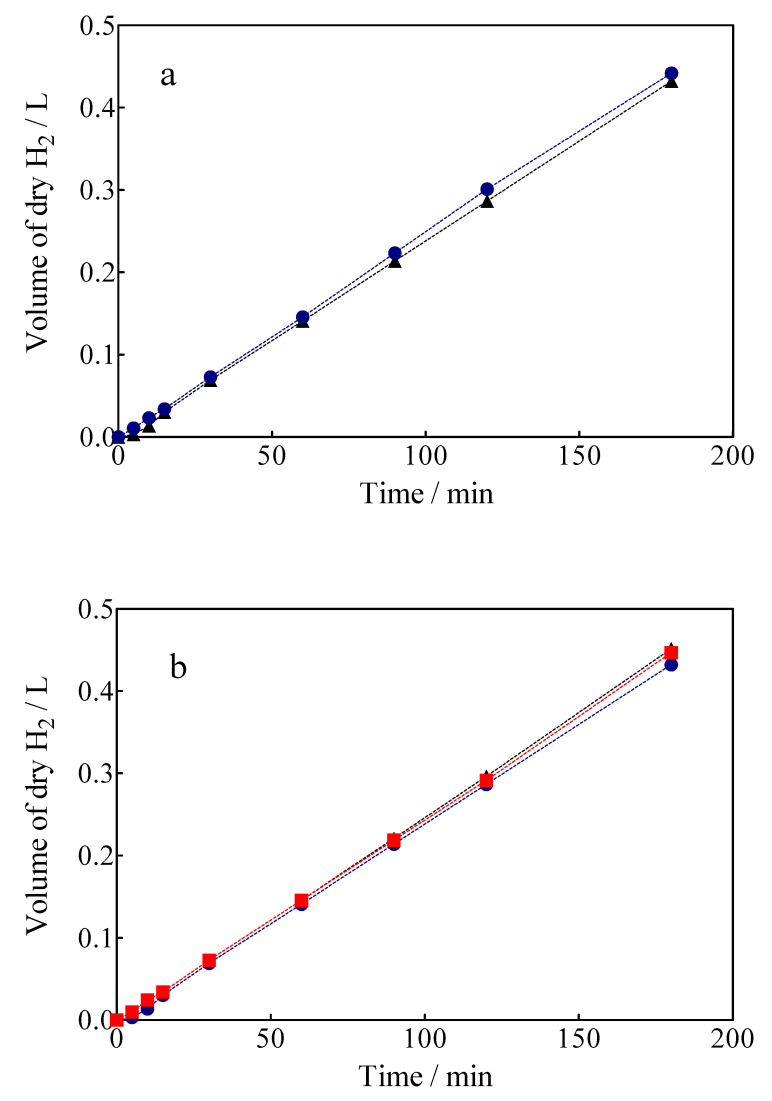
The amount of H_2_ obtained *vs* electrolysis time by applying 15 mA cm^−2^: (**a**) 0.05M Na_2_SO_4_ + 20 mg L^−1^ (black circle) or 50 mg L^−1^ (blue circle) of Calcon dye, (**b**) different flow rate in the anodic compartment ((●) 10, (■) 39 and (▲) 90 mL min^−1^).

The volume of green H_2_ produced was corrected by the quantity of water vapor generated in the gas collector due to the H_2_ evolution on the cathode. For comparison, the experimental values are shown against the corresponding estimated values concerning the volume of H_2_. As can be observed in Figure 10, under galvanostatic conditions, the measured volume of green H_2_ produced during the EO of a model organic compound varied linearly as a function of electrolysis time, showing in all experimental cases a correlation factor of R^2^ > 0.995. This is just as predicted by Equation (7) and in good agreement with Faraday’s law. Indeed, Equation (7) establishes that the production of H_2_ by the traditional electrolysis of water only depends on the intensity of the applied current, but in the electrolysis processes of organic sacrificial compounds (OSC), it is important to verify if variables such as the concentration of OSC or anolyte flow rate may or not influence the amount of H_2_ produced. Therefore, an additional set of experiments was carried out varying the initial concentration of Calcon from 20 to 50 mg L^−1^ (Figure 11a), and with an anolyte flow through the anode compartment of 10, 39 and 90 mL min^−1^ (Figure 11b).

As can be observed in Figure 11a, the H_2_ production volumes obtained when concentrations of 20 and 50 mg L^−1^ of Calcon dye as OSC were about ±0.431 L after 180 min of electrolysis. The results showed that the increase of the concentration of Calcon slightly influenced the volume of H_2_ produced. The similarity in the H_2_ amount production was attained until 40–50 min; after that, the production of H_2_ slightly increased until the end of the electrolysis, when the pollutant concentration passed from 20 to 50 mg L^−1^. Meanwhile, the TOC removal efficiencies obtained at the anodic compartment were about 42 and 38%, respectively, which confirmed the viability of the hybrid process. In this case, the production of heterogeneous free ^•^OH at BDD surface promotes the indirect oxidation of Calcon, even when direct oxidation can be simultaneously attained as previously described by electrochemical measurements. In the latter, direct interactions between the Calcon chemical structure and diamond surface should be attained. Conversely, the efficacy of the process could be also affected by the mass transport conditions as well as the oxygen evolution reaction (the waste reaction of the hydroxyl radicals), in the former approach. Another feature that could be considered is that the elimination of organic matter was efficiently achieved during the first 40–50 min of electrolysis in all cases; after that, the process was dependent on the mass transport conditions [63]. At this point, the H^+^ produced via the electrochemical mineralization of small organic intermediates (such as carboxylic acids) and/or the electrolysis of the supporting electrolyte (e.g.: H_2_O and H_2_SO_4_) at the anodic compartment could be transported to the cathodic reservoir, participating in a slight improvement in H_2_ production (see Figure 11a). Therefore, the effects that could be associated with the flow rate in the anodic compartment, where the hydrodynamic conditions applied are generally responsible for improving the mass transport towards the anode and promoting a faster reaction between the OSC and the oxidizing agents at the BDD surface [62], should be also considered. 

Under different flow rate conditions at the anode compartment (10, 39 and 90 mL min^−1^) (see Figure 11b), it is possible to observe that a slight influence on the volume of H_2_ produced was achieved at the cathodic compartment, while the mineralization efficiencies have been significantly influenced (31% at 10 mL min^−1^, 42% at 39 mL min^−1^ and 49% at 90 mL min^−1^). This result clearly showed that the protons (H^+^) involved in the formation of the green H_2_ on the cathode surface are not only those released by the oxidation of the organic molecule (Equation (4)), which are transferred to the cathode through the membrane of Nafion^®^-350, but also those generated by the discharge of water on the anode to form ^•^OH radicals (H_2_O → 2H^+^ + ½ O_2_), which are also transferred to the cathode by the same transport mechanism. In this sense, the cathode will only act as a proton sink given the electrolysis conditions, because its chemical nature will not have a significant influence on the H_2_ evolution reaction (HER), since the potential difference established at the intensities of applied current exceeds the overpotential required for the HER [33]. Therefore, the hybrid approach is sustainable because, on the one hand, the decontamination of water is attained (in meeting SDG 6), and on the other hand, the electrochemical production of a high added-value product (green H_2_) is also achieved (in meeting SDG 7) by exploitation of the electrical potential for the former process. 

**Figure 12:**
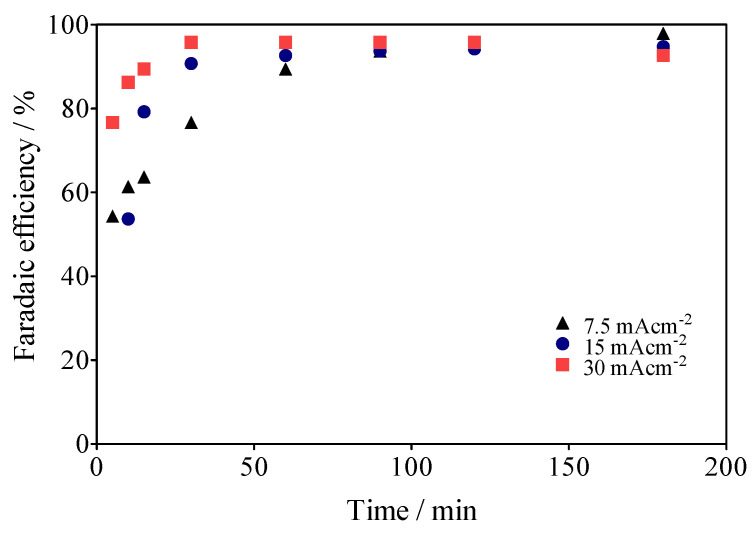
Faradaic efficiencies (in %) for data collected about the volume of H_2_ in Figure 7.

Within this framework, the reduction of operating costs of the electrochemical system in this hybrid approach, the use of SS (Ni-Fe base) as a cathode material, instead of Pt [64,65,66], which is considered the most efficient material for H_2_ production, was incentivized. It should be noted that the cathode was used in two forms: SS-316 plate, and SS mesh (Ni-Fe)-based. Moreover, it was observed that at 15 mA cm^−2^, for example, the green H_2_ production was slightly higher on the SS-mesh (2.4 mL min^−1^) than on the SS-plate (1.4 mL min^−1^), which is a consequence of a better release of H_2_ bubbles in the SS-mesh, compared to the SS-plate, which retains most of the bubbles on its surface. Moreover, other intrinsic factors to the HER process [66], such as cell design, electrode spacing, use of separation membranes between compartments, and even the appearance of parasitic currents [67] can influence both the volume of H_2_ produced and its Faradaic efficiency (FE). Taking into account the previous considerations, the FE was estimated according to Equation (8):

(8) $$FE=\frac{\left(V_{H_{2}}\right)\left(\alpha \right)\left(F\right)}{\left(24.45\right)\left(i\right)\left(t\right)}$$

where *V_H2_* is the volume of H_2_ collected at the time *t*, *α* is the number of electrons transferred (2 for H_2_), *F* is the Faraday constant (96,487 C/mole), 24.45 is the molar volume of a gas at 298.15 K at 1 atm, *i* is the current intensity, and t is the electrolysis time. As can be observed in Figure 12, the real Faradaic efficiency (rFE) varied at the first 40–50 min of the electrolysis and remained above 90% until the end of the process. This behavior is due to the use of the electrical charge for anodic reactions and the existence of the fuel crossover and internal currents phenomena [68] in the former stage, which is a typical effect. Meanwhile, in the latter stage, the existence of the intrinsic factors affected the rFE, but above all because the electrochemical system is also operated by means of the impulse of an alternate source of renewable energy, such as the photovoltaic arrangement shown in Figure 1.

This last consideration is important in view of the economic/environmental sustainability of the system studied here, since it demonstrates that the use of renewable energies associated with electrochemical systems for advanced oxidation processes (AOPs) is a feasible hybrid approach to produce high added-value products, such as green H_2_. Therefore, both processes (depuration of water and energy vector production) are viable at costs of a fraction of traditional systems.

### 3.5. Specific Energy Consumption for the Green H_2_ Production

**Figure 13:**
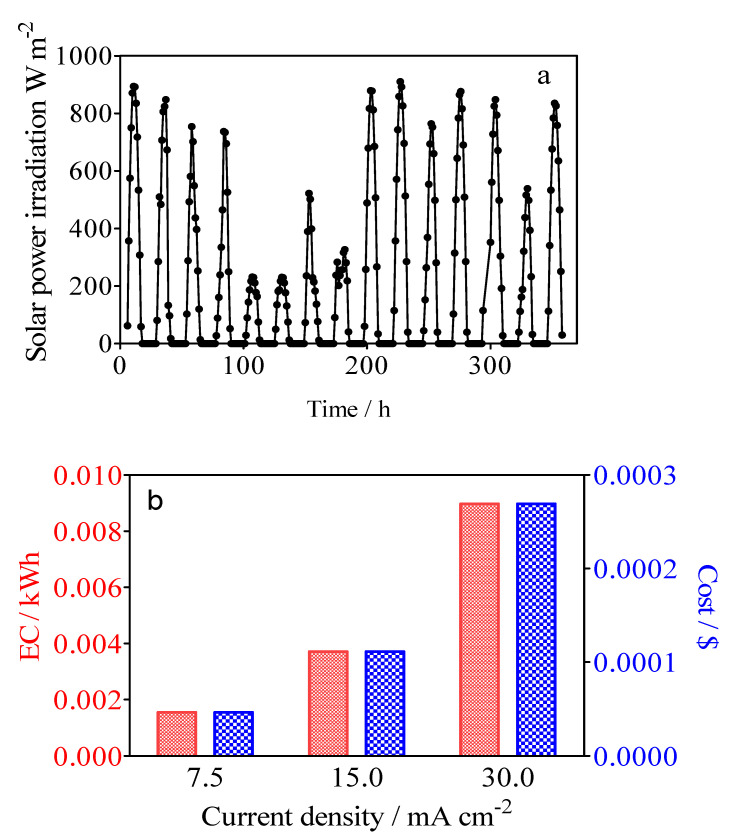
Changes in (**a**) the solar power irradiation intensity and (**b**) the effect of applied current densities by using BDD/SS (Calcon solution 20 mg L^−1^ in 0.25 M H_2_SO_4_, and the catholyte solution of 0.25 M H_2_SO_4_) on the electrical energy consumption of the photovoltaic system for H_2_ production in red, while the cost of the process was estimated based on the International Renewable Energy Agency.

Figure 13a shows the solar power irradiation intensity during the 15-day experimental test developed. Solar global irradiation in Natal obtained corresponded to 72,508 W/m^2^, which coincide with the sum of all 15 days. As can be observed in Figure 13a, on the one hand, the solar radiation ranged from average values of 217 to 917 W/m^2^ on sunny and clear days, while on the other hand, radiation values were minimum on cloudy days, leading to values ranging from 106 to 130 W/m^2^. It is important to consider that the ambient temperature varied from a maximum of 28 °C on cloudy days to 34 °C on summer (high temperature) days, corresponding to tropical weather. As indicated above, the irradiation increased gradually from an early hour (5:00 a.m.), which corresponds to the rise in solar irradiation intensity and daily peak of 18.2 A which lasts for approximately 4 h, and which was observed starting from around noon. The photovoltaic system was able to generate current intensities of over 10 A for 8–9 h/day when the irradiation intensity was greater than 200 W/m^2^, which contributed to the constant voltage of ~27 V recorded from the photovoltaic modules to the batteries [69]. This information shows the potential for green water remediation and green H_2_ production using an off-grid photovoltaic system as an application of remote devices in areas of high solar irradiation resource [70,71]. 

The electrical energy consumption of the photovoltaic system for H_2_ production is obtained to according to Equation (9):

(9) $$E_{s}=\frac{E_{stack}\times I_{stack}\times t}{VH_{2}}$$

where *E_s_* is the specific energy consumption, *I_stack_* is the stack current, *E_stack_* is the stack voltage, *mH_2_* is the H_2_ mass flow rate (m^3^), and *t* (h) is the time. Thus, the energy consumption defines the amount of energy consumed to produce a mass unit of H_2_. Figure 13b shows the values for the different current densities of 6.94 kWh m^−3^ H_2_, 8.81 kWh m^−3^ H_2_ and 11.12 kWh m^−3^ H_2_, respectively. As was expected, the increase of current density produces an increase in energy consumption. Another important piece of information is that the energy cost ($) after 3 h of electrolysis for the simultaneous removal of organic pollutant and green H_2_ using a photovoltaic system, based on the International Renewable Energy Agency (IRENA) costs, was estimated (1 kW h = USD 0.0057 (IRENA website)), achieving about USD 0.0395, 0.0502 and 0.0634 m^−3^ for H_2_ production at 7.5, 15 and 30 mA cm^−2^, respectively [72]. The average residential electricity rate in the U.S. is USD 0.17 per kWh, and consequently a photovoltaic system is relatively cheap compared to conventional electricity. It is important to consider that the electrical energy produced that was not used for the hybrid process was stored in the battery system, but it was not considered as a positive cost value where the excess could be injected into the electrical grid. Additionally, the green H_2_ production should be economically evaluated in different forms according to the process in which it was produced and its kind, such as green, blue, or grey. Therefore, the positive cost, via the sale of the green H_2_ production, should be considered in the total techno-economic analysis. However, it will be estimated and reported in a further paper.

## 4. Conclusions

It was demonstrated in this study that: (i) the development of a hybrid process for removing organic matter in a effluent and producing green H_2_ simultaneously is a feasible water-energy environmental alternative with higher efficiencies and reasonable cost requirements to fulfill SDG 6 and 7; (ii) water depollution and green H_2_ production was always improved when an increase in the applied current density was attained; (iii) the effectiveness of the water anodic electrochemical-based solution in the hybrid process mainly depended on the electrogeneration of oxidizing species on a diamond electrode (^•^OH, SO_4_^−•^ and S_2_O_8_^2−^), which promoted the indirect oxidation of Calcon dye at higher applied current densities; (iv) direct oxidation on the BDD surface is possible at lower applied current densities in the hybrid process, confirming the versatile oxidation behavior of diamond films; (v) in the cathodic reaction of the hybrid process, the use of SS proved to be efficient for H_2_ production, with the advantage of having a lower cost compared to other commonly used cathodes for H_2_ generation, such as Pt; and (vi) economic and environmental advantages have been attributed to the wastewater treatment, the valuable byproduct produced (green H_2)_, and when combined with solar energy, electrochemical wastewater processing can become energy-efficient and cost-effective.

Another investigation that should be carried out is related to the relationship of the H^+^ concentration produced via the electrochemical mineralization of small organic intermediates (such as carboxylic acids), and/or the electrolysis of the supporting electrolyte (e.g., H_2_O and H_2_SO_4_) at the anodic compartment, which could be transported to the cathodic reservoir, participating in the enhancement of H_2_ production as well as the coexistence of the parallel effects, due to the electrical charge for anodic reactions influencing H_2_ production and the fuel crossover and internal currents phenomena.

## Figures and Tables

**Table 1:** Chemical structure and characteristics of the azo dye used in this work (Eriochrome Blue Black R).

Chemical Structure	** 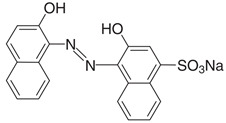 **
Chemical formula	C_20_H_13_N_2_NaO_5_S
IUPAC name	1-(2-Hydroxy-1-naphthylazo)-2-naphthol-4-sulfonic acid sodium salt
Synonym	Eriochrome Blue Black R; Mordant Black 17; Palatine Chrome Black 6BN; Anthracene blue black; Alizarin blue black BG
Commercial name	Calcon
Color Index number	15,705
Molecular Weight (g mol^−1^)	416.38
λ_máx_ (nm)	513
Solubility in water	20 mg mL^−1^
Purity	98%
CAS number:	2538-85-4

## Data Availability

Not applicable.

## Supplementary Materials

The following supporting information can be downloaded at: https://www.mdpi.com/article/10.3390/ma15217445/s1, Figure S1. Energy dispersive X-ray spectroscopy. Inset: Scanning Electron Microscopy of the mesh (Ni-Fe base) cathode. Figure S2. FTIR spectra of Calcon dye before and after electrolysis. The samples were chosen at the best experimental condition using Calcon 20 mg L^−1^ in 0.25 M H_2_SO_4_ at a current density of 30 mA cm^−2^. (*) identification for the more important signals. Figure S3. Profiles of evolution of short-chain carboxylic acids (tartaric, oxalic, acetic, and formic acids) during the electrolysis of Calcon 20 mg L^−1^ in 0.25 M H_2_SO_4_ at a current density of 30 mA cm^−2^.
