# Supplementary material for: Photovoltaic Electrochemically Driven Degradation of Calcon Dye with Simultaneous Green Hydrogen Production
Source: Materials (Basel). 2022 Oct 24;15(21):7445. doi: 10.3390/ma15217445 (PMC9655423; doi:10.3390/ma15217445)
Supplement: Supplementary file 1 [file materials-15-07445-s001.zip › materials-1880296-supplementary.pdf]

Supplementary material

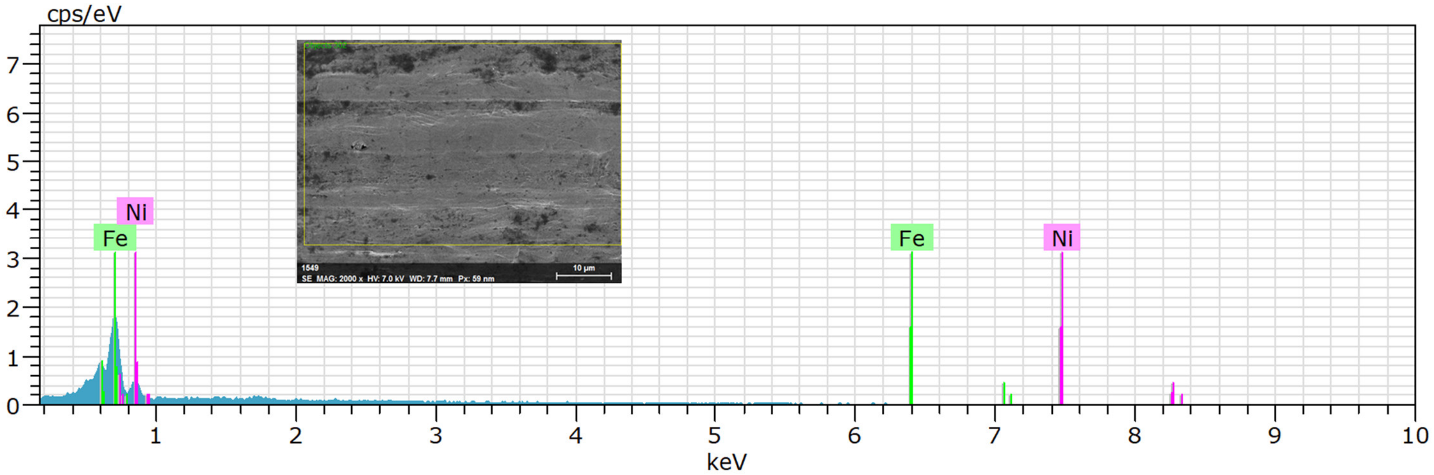

Figure S1. Energy dispersive X-ray spectroscopy. Inset: Scanning Electron Microscopy of the mesh (Ni-Fe base) cathode.

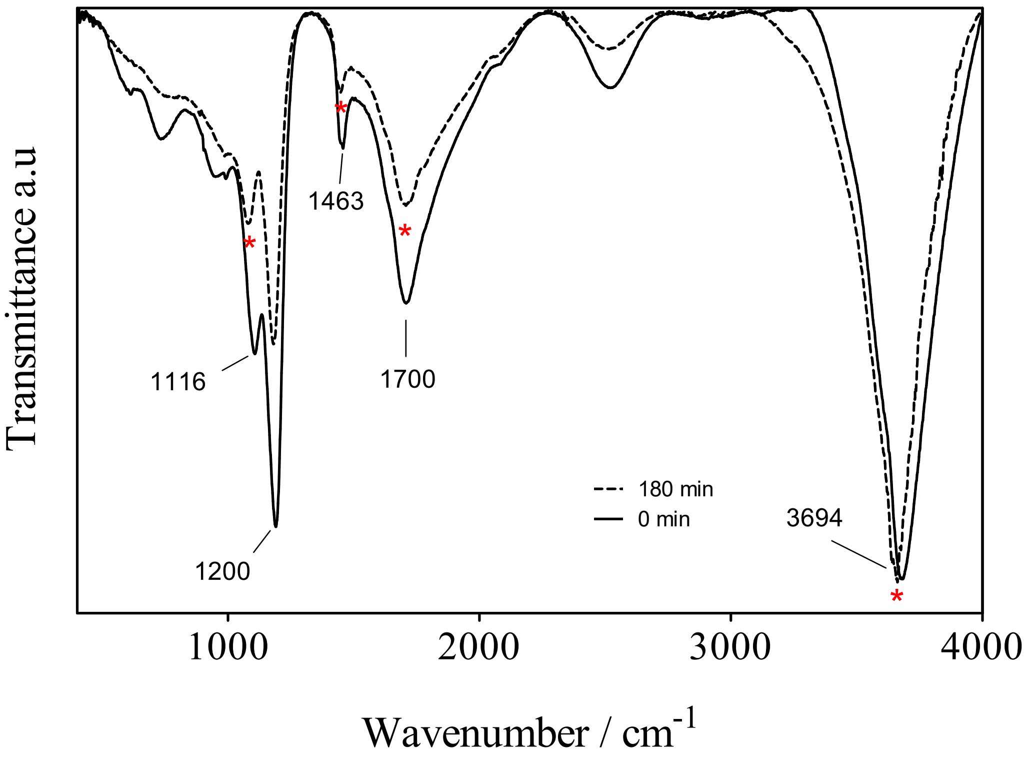

Figure S2. FTIR spectra of Calcon dye before and after electrolysis. The samples were chosen at the best experimental condition using Calcon 20 mg L^{-1} in 0.25 M H_2SO_4 at a current density of 30 mA cm^{-2}. (*) identification for the most important signals.

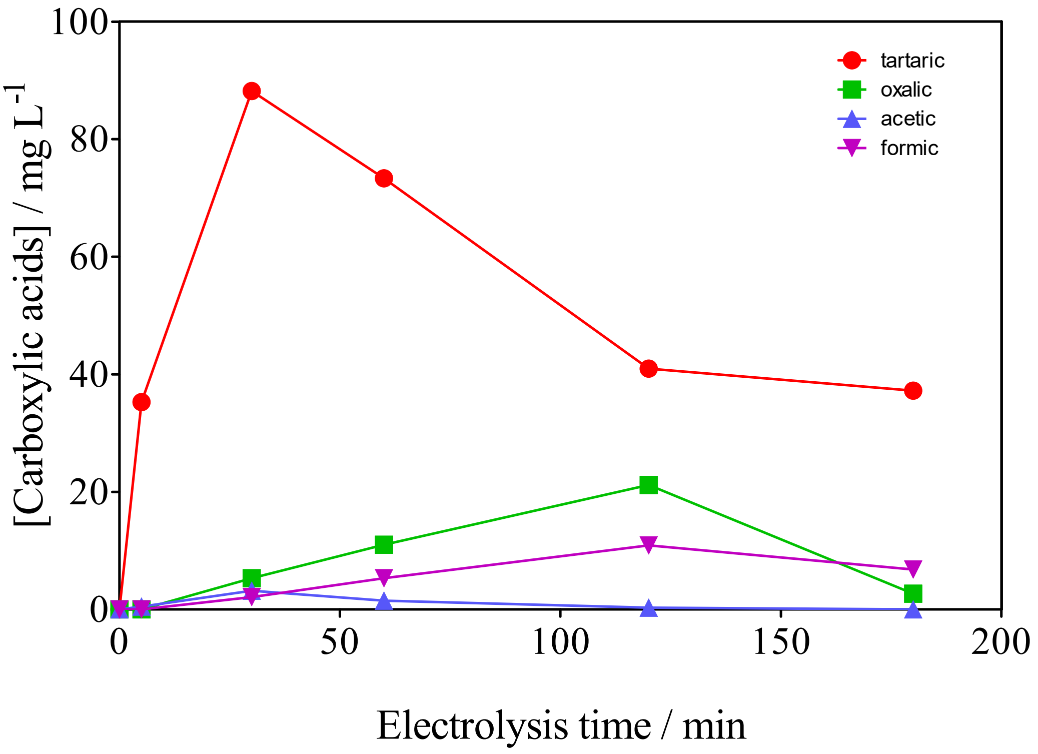

Figure S3. Profiles of evolution of short-chain carboxylic acids (tartaric, oxalic, acetic and formic acids) during the electrolysis of Calcon 20 mg L^{-1} in 0.25 M H_2SO_4 at a current density of 30 mA cm^{-2}.
